# Supplementary material for: Genomic Characterization of Salmonella typhimurium DT104 Strains Associated with Cattle and Beef Products
Source: Pathogens. 2021 Apr 27;10(5):529. doi: 10.3390/pathogens10050529 (PMC8145149; doi:10.3390/pathogens10050529)
Supplement: Supplementary file 1 [file pathogens-10-00529-s001.zip › Supplemental Figures.pdf]

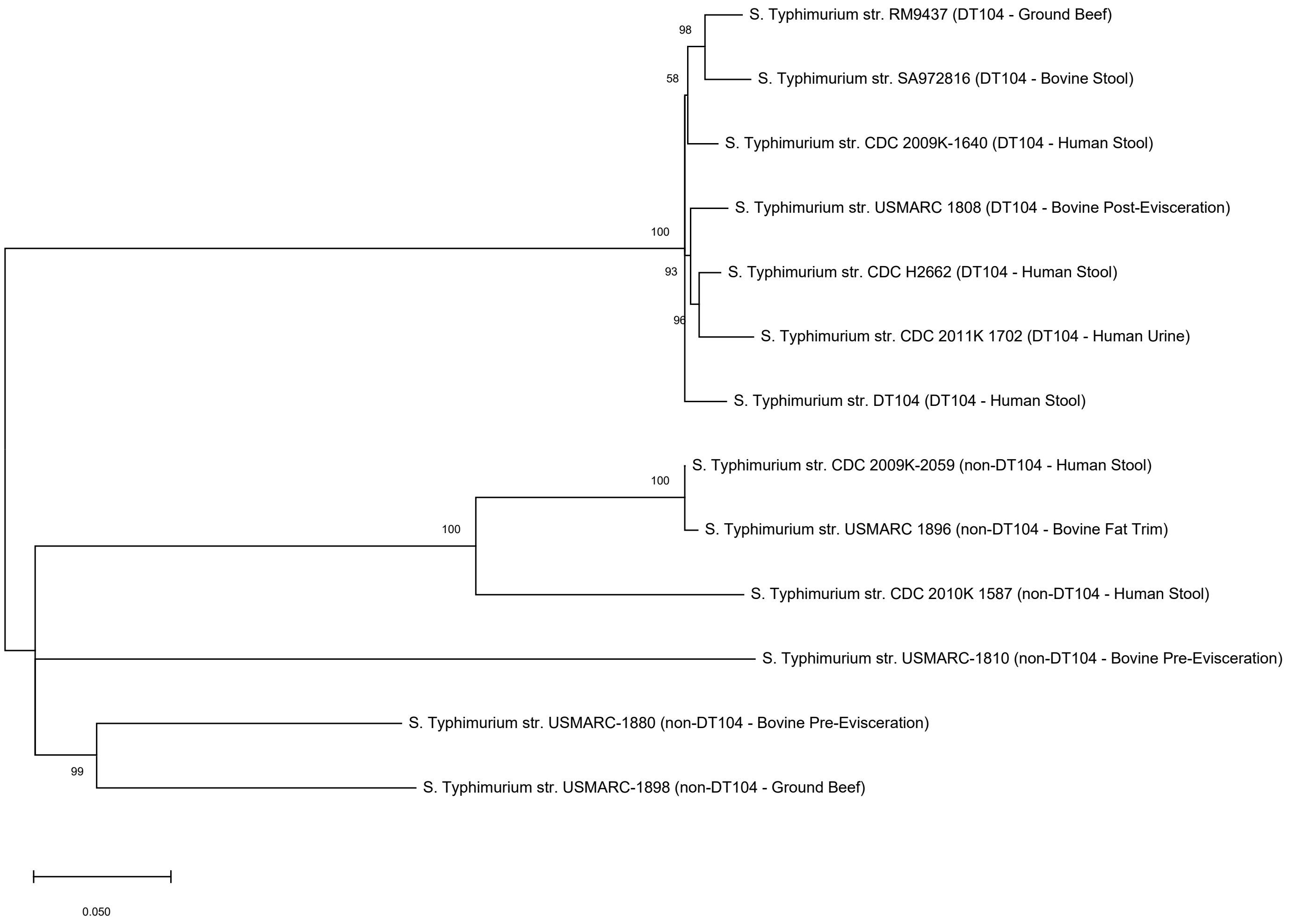

Supplemental Figure 1. Phylogenetic analysis of thirteen ST19 *S.* Typhimurium strains associated with either clinical or bovine sources. Maximum-likelihood tree generated using the Tamura-Nei model with gamma distribution with 1,000 pseudoreplicates involving a dataset of 1,781 filtered high-quality SNPs between the strains. Scale bar represents number of substitutions per site.

A.

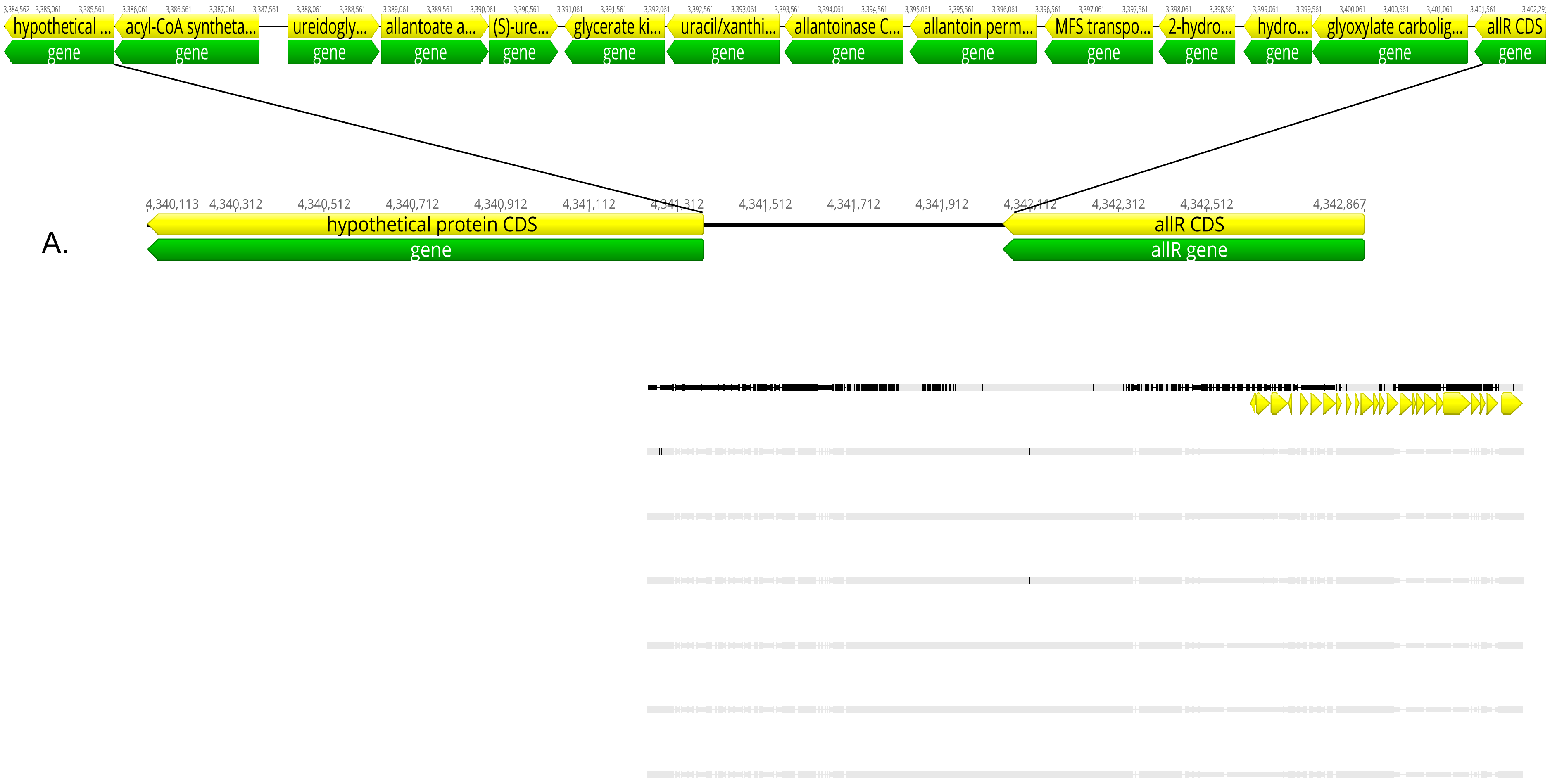

Supplemental Figure 2. Genetic variation of DT104 strains compared to non-DT104 strains of the ST19 lineage. A. Deletion of allantoin utilization pathway in DT104 strains versus non-DT104 strains. *S.* Typhimurium str. CDC 2010K-1587 is the non-DT104 strain on top that contains the genes for the allantoin utilization pathway, and *S.* Typhimurium str. DT104 is the DT104 strain on the bottom showing the deletion of the genes. B. Alignment of *Enterobacteria* phage ST104 prophage between DT104 strains and *S.* Typhimurium str. CDC 2010K-1587, the only non-D104 strain to contain prophage, which demonstrates quite a bit of diversity between ST104 prophage in the non-DT104 strain versus those present in the DT104 strains.
