# Supplementary material for: Genomic Characterization of Salmonella typhimurium DT104 Strains Associated with Cattle and Beef Products
Source: Pathogens. 2021 Apr 27;10(5):529. doi: 10.3390/pathogens10050529 (PMC8145149; doi:10.3390/pathogens10050529)
Supplement: Supplementary file 1 [file pathogens-10-00529-s001.zip › Supplemental Table 3 - Core genes.pdf]

Supplemental Table 3. Core genes

	Core genes[1]	Shell[2]	Cloud[3]
All strains	4,300	877	587
DT104 strains	4,512	300	49
Non-DT104 strains	4,331	1,232	0
Bovine strains	4,308	586	698
Clinical strains	4,350	968	0

[1] All strains: 12<=strains<=13; DT104 and bovine strains: 6<=strains<=7; non-DT104 and clinical strains: 5<=strains<=6; 6<=strains<=7

[2] All strains: 1<=strains<12; DT104 and bovine strains: 1<=strains<6; non-DT104 and clinical strains: 0<=strains<5

[3] All, DT104, non-DT104, bovine and clinical strains: strains<1
